# Virtual Reality Training Affects Center of Pressure (COP)-Based Balance Parameters in Older Individuals

**DOI:** 10.3390/app14167182

**Published:** 2024-08-15

**Authors:** Nicole Arnold, Oshin Wilson, Lara Thompson

**Affiliations:** Center for Biomechanical & Rehabilitation Engineering, Biomedical Engineering Program, School of Engineering and Applied Sciences, University of the District of Columbia, 4200 Connecticut Ave. NW, Washington, DC 20008, USA

**Keywords:** aging, elderly, virtual reality, balance training, center of pressure, falls

## Abstract

Postural imbalance is a leading cause of injury in older adults. Our study investigated the effectiveness of virtual reality (VR)-based interventions on balance ability in this population. Here, we examined 21 older, healthy adults (75.8 ± 5.2 years old). Participants performed 6 weeks of balance training, twice per week for 30 min; the experimental group donned an Oculus VR headset during the training while control participants did not. To assess balance ability, a force platform measured displacement of the center of pressure (COP) during quiet standing in double-leg, tandem, and single-leg stances with eyes closed pre- and post-assessment. COP measurements included mediolateral (ML) and anterior–posterior (AP) directions for root mean square (RMS), peak-to-peak displacement (MAXD), total excursion (TE), and 95% confidence area ellipse (AE) parameters. Post-training assessments showed improvements (significant decreases) in the COP parameters. Control group COP parameters improved in various stances ranging from a 3% to 40% decrease on average. The VR group improved MAXD, TE, and 95% AE ranging from a 5% to 47% decrease, on average, across various stances post-compared to pre-training. VR-based exercise training programs may encourage older adults to engage in mobility exercises, leading to a reduced risk of falls or injuries.

## 1. Introduction

As the population ages, falls and injuries in the elderly are a major concern leading to decreased quality of life and thus the inability to care for oneself. According to the Center for Disease Control (CDC), nearly 36 million older adults fall each year [1]. Nearly 3 million older adults were treated in emergency room departments for a fall-related injury [2,3]. Falls are the leading cause of fatal and nonfatal injuries among persons aged over 65 years old [1]. Aging has proven to be correlated with an increase in the severity and the number of injuries [4].

Several risk factors have been identified that may lead to falls in the elderly. Postural instability when one’s base of support has been reduced was identified as a major intrinsic risk factor for the older population [5]. Falls have been linked to a reduction in static balance measured by postural sway during various foot placements [6,7]. Additionally, some studies have reported lack of exercise and increased fall risk for older adults [8,9]. There have been several traditional programs (such as resistance training and balance exercises) aimed to increase mobility, posture, and balance to reduce falls in the elderly [8–11]. Group and home-based exercise programs with various tasks significantly reduced the risk of falls of those at both high and low risk [5,6,8,9,12]. These methods have proven somewhat effective in that there are still issues with compliance and realistic testing environments.

Virtual reality (VR) training has the capability to provide a safe environment that can create scenarios that simulate real life. Mobility and balance practice using a VR headset may provide an avenue for accessible, effective home-based balance training. Additionally, other external settings, such as rehabilitation facilities, senior centers, or nursing homes may also benefit. More recently, researchers have begun to use VR headsets to explore their impact on balance and falls in elderly adults [13–17]. Several studies have displayed that VR training increases the possibilities of motor training and can help reduce the risk of falls by improving the static and dynamic balance [11,17].

A key component to assess those at risk for falls must require the proper and necessary tools that accurately mimic and measure balance performance. In biomechanics research, force plates are instrumental devices that provide a detailed understanding of external forces acting against the body. These devices have provided meaningful insights in terms of clinical evaluations and have been used to assess sports performance, injury recovery, and rehabilitation. Investigation of balance ability has been characterized with measures created by the displacement from the center of pressure (COP). The COP is measured with a force platform, for example, while the participant performs quiet standing in double-leg, tandem, and single-leg stances. Balance control during these stances has been analyzed with the trajectory of the COP, or COP displacement time series. The COP displacement time series reflects the movement of the point location of the vertical ground reaction force vector; this vector represents the weighted average of all pressures of the surface area in contact with the support surface (ground). More commonly, studies have considered balance stability with the root mean square (RMS) of the medio-lateral (ML) and anterior–posterior (AP) COP displacement. Other measures include peak-to-peak displacement (MAXD) (i.e., maximum displacement in the ML and AP directions), total excursion (TE) (i.e., the total length of the COP path), and 95% confidence area (i.e., area which approximately 95% of the points on the COP path occupy) extracted from the COP measurements captured [18]. Evaluation of COP measurements will be useful to researchers and clinical staff to determine age-related changes in balance ability tied to VR training, in that previous VR balance training studies have focused their evaluation of performance predominantly on more qualitative measures of balance such as the Timed Up and Go (TUG) [19–21]. Thus, the purpose of this study was to investigate the effect of VR-based training on the balance ability of healthy older adults before and after an exercise training program via COP-based parameters. Here, we hypothesized that several weeks of VR training could impact balance and falls observable by the COP.

## 2. Materials and Methods

### 2.1. Study Overview

**Figure 1.**
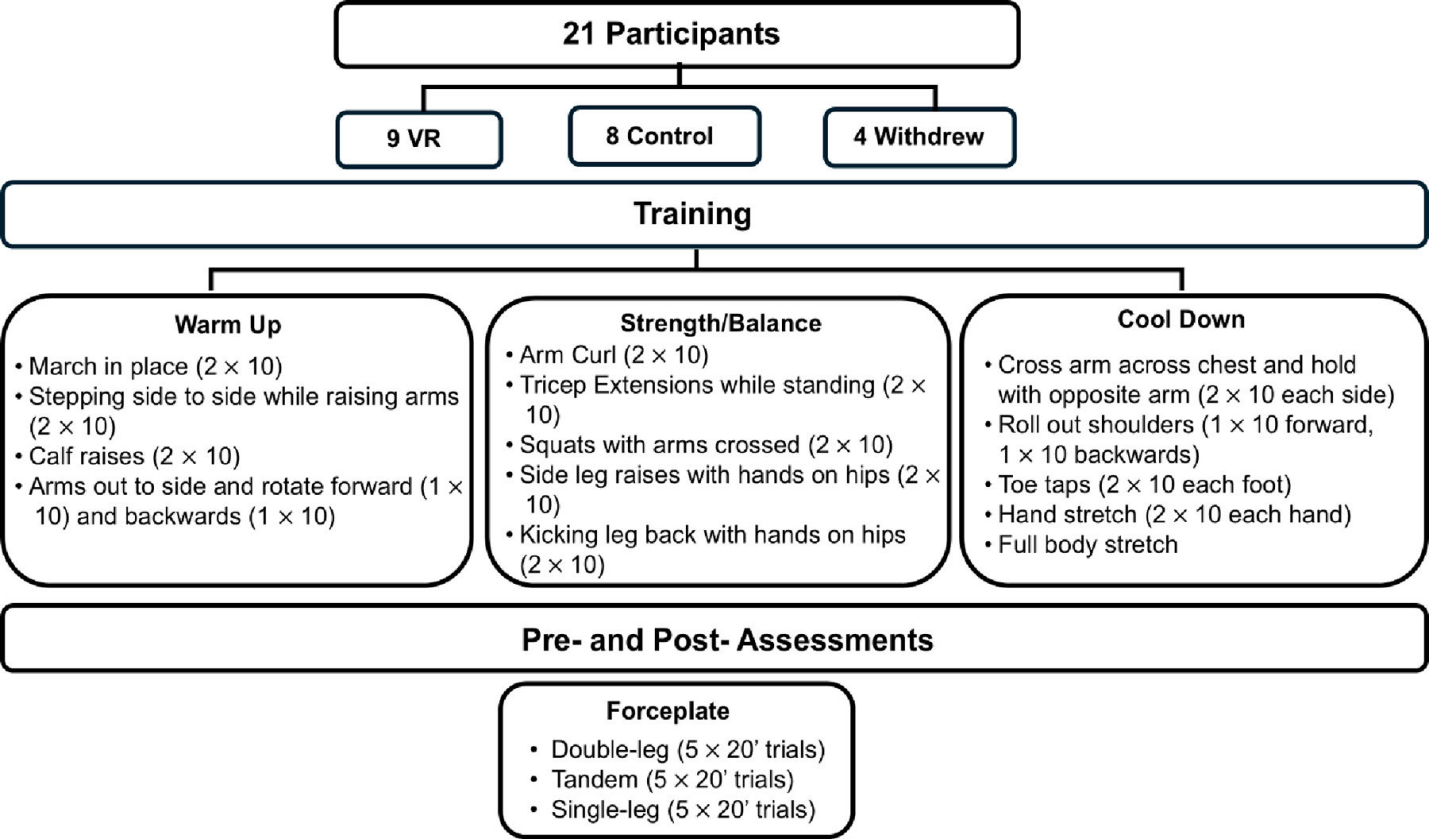
Overview of research study.

Research was conducted at the University of the District of Columbia in the Center for Biomechanical & Rehabilitation Engineering. This study was approved by the IRB (#2073871-1) and all participants signed and consented to the research study. Figure 1 details the outline of this study.

#### 2.1.1. Participants

Participant inclusion criteria required individuals to be between 60- and 85-year-old males and females, have the capacity to ambulate unassisted, be free from any medical condition or physical disability, demonstrate adequate cognitive ability (i.e., to score within the normal range as measured by the Mini Mental State Examination (MMSE)), have adequate vision and hearing, and no history of a fall in the past year. Exclusion criteria were individuals with significant medical issues, serious lower limb discomfort, and incapacity of walking without aid. There were two groups of participants: (1) the control group that performed training without the VR headset and (2) the experimental, or VR, group, that performed the training with the VR headset. A total of 21 participants, including 3 males and 18 females, participated in this study; however, 4 withdrew. Participant withdrawal from the study was due to scheduling and prior commitments tied to travel and other engagements. This resulted in 9 participants (75.9 ± 3.7 years old) in the VR group and 8 participants (75.1 ± 6.7 years old) in the control group. A demographic overview of each patient is shown in Table 1, as in [13].

#### 2.1.2. Training

**Figure 2.**
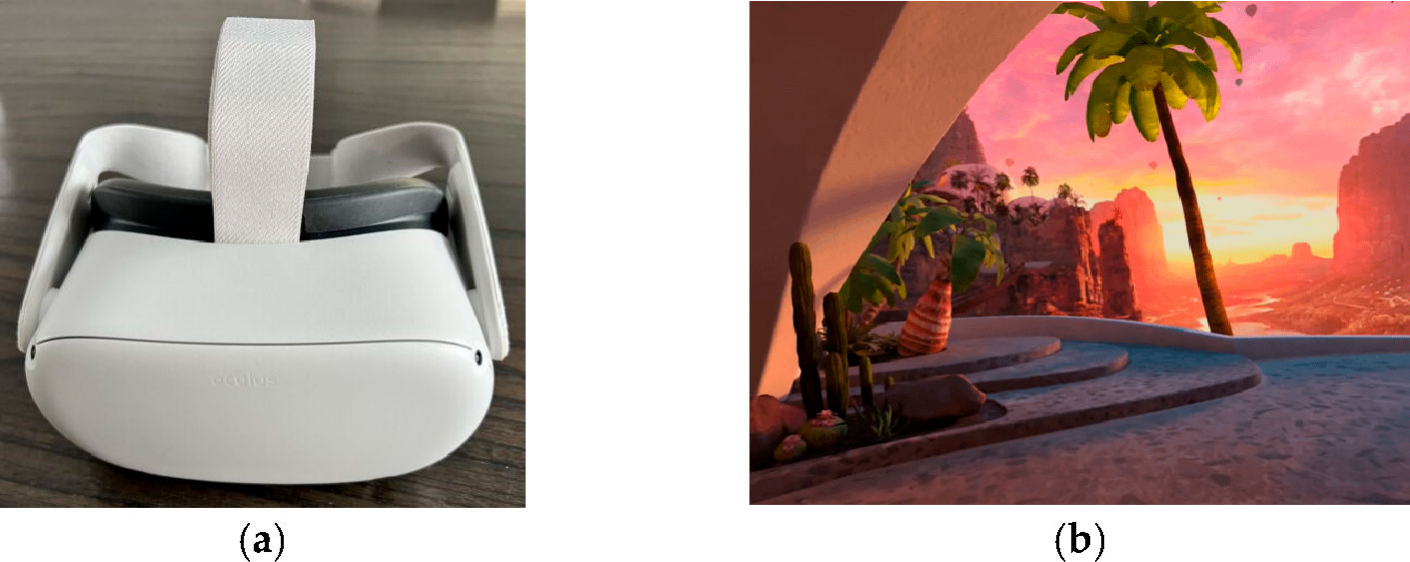
(**a**) Oculus VR headset and (**b**) scene environment seen by participants in the VR group.

The Oculus VR system (Figure 2a) was used during our training of the older participants within the VR group. The VR system provided a safe and controlled environment (scene shown in Figure 2b) for the participants to practice different strength and balance exercises.

Exercise training included two 30 min sessions per week for six weeks (or 12 training sessions), with specific exercises targeting the characteristics of balance, such as static and dynamic balance, sensory incorporation, and motor coordination (summary in middle row of Figure 1 for both groups). The training exercise program was modified from a workout created by the National Institute on Aging geared toward older adult balance exercises [22]. These exercises included a warm-up designed to prime the muscles for physical activity, followed by exercises composed of balancing tasks, increasing in complexity while also challening the particpants’ strength (e.g., body weight squats) and balance (e.g., side leg raises). Finally, the training concluded with a cool down and flexibility movements (e.g., full body stretch). Additionally, for the first 3 weeks of training participants performed the list of exercises shown in Figure 1 without weights. For the second 3 weeks of training, participants performed the same exercises with an additional set (three sets instead of two sets) and wore 1 lb wrists weights on each wrist.

#### 2.1.3. Pre- and Post-Training Assessments

**Figure 3.**
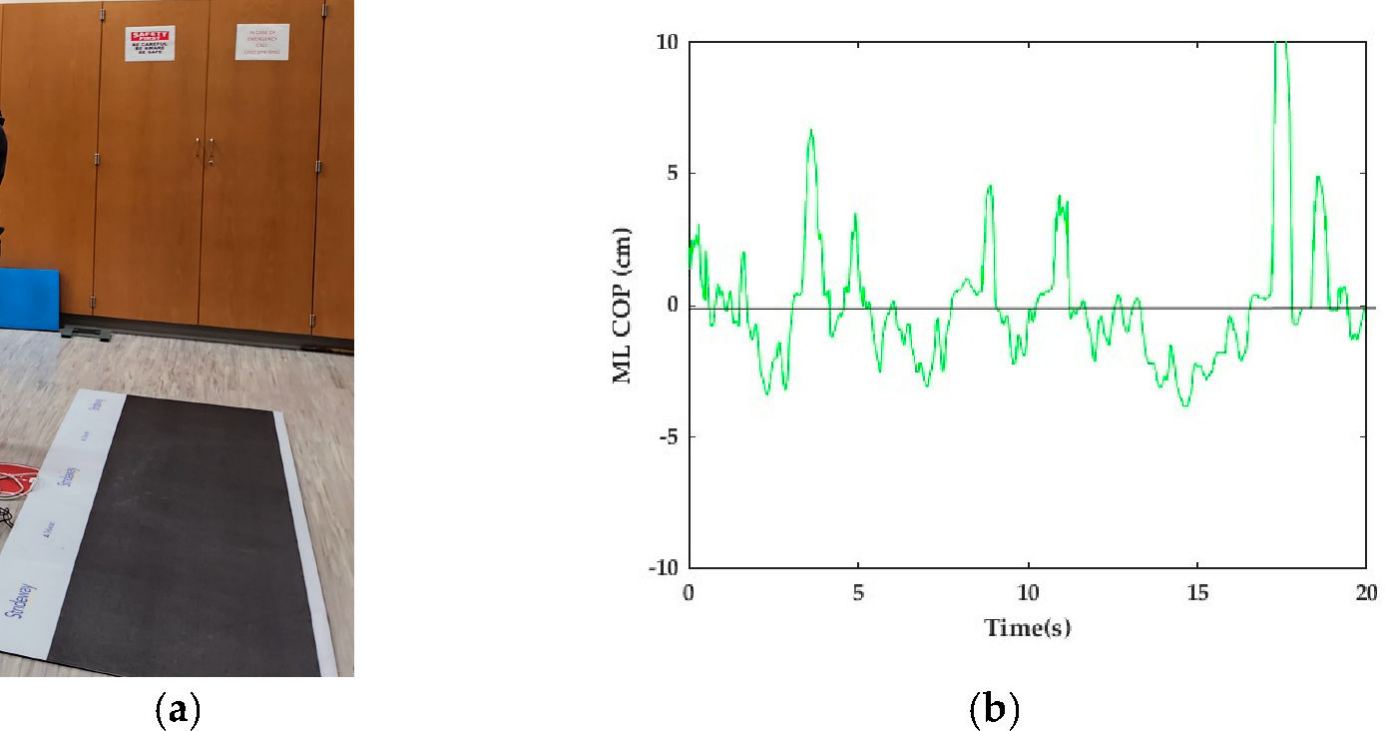
(**a**) Force plate used to acquire COP data and (**b**) example of ML COP time series.

Participants completed a pre-assessment, 6-week training (12 training sessions), and a post-assessment. To measure center of pressure (COP), a force plate (Tekscan, Norwood, MA, USA) (Figure 3a) was used to obtain COP data at 50 Hz (Figure 3b). To acquire the COP time series, stationary (standing) balance was tested, in which participants performed five 20 second trials of a double-leg stance (i.e., feet should width apart), tandem stance (i.e., heel of one foot directly in front of the toes of the other foot), and single-leg stance (standing on one leg) on the force plate with eyes closed (Figure 3a).

Though not presented here, other balance assessments included standing balance and mobility using the Balance Error Scoring System (BESS) [23] and Timed Up and Go (TUG) test [19], respectively. Also not reported, but assessed, were the psychological and emotional aspects of balance and falling, via the Activities-specific Balance Confidence [24] scale, the Tinetti Falls Efficacy Scale (FES) [25], and the Geriatric Depression Scale (GDS) [26]. These measurements have commonly been used in other research-related studies, rehabilitation, and concussion assessments to monitor changes in balance and the efficacy of training programs and have been reported by us in a previous publication [13]. However, here we highlight and discuss only force data metrics tied to the COP that were used to assess standing balance.

### 2.2. Analysis

#### 2.2.1. COP Parameters

The COP data were first detrended (i.e., removing the COP time series offset displacement (Equations (1) and (2)). Using the detrended data, COP parameters were calculated (Equations (3)–(11)). The COP metrics computed from this zero-mean time series were the root mean square (RMS) and peak-to-peak displacement (MAXD) (i.e., RMS and MAXD for both the anteroposterior (AP) and mediolateral (ML) directions), total excursion (TE) and 95% area ellipse (AE), shown in Equations (3)–(11), respectively [18]. A custom MATLAB program was written to compute these parameters (Mathworks, Inc., Version 2023a, Natick, MA, USA). Further, parameters were collected and calculated for both pre- and post-assessment trials. In each equation, N is the number of data points over 20 second trials.

AP COP time series without offset

(1) $$AP={AP}_{o}-\frac{1}{N}\sum \;|AP[n]|$$

ML COP time series without offset

(2) $$ML=ML_{o}-\frac{1}{N}\sum \;|ML[n]|$$

where ${AP}_{\circ }$ or ${ML}_{\circ }$ are the AP or ML COP path relative to the origin of the force platform, respectively, and N is the number of data points.

AP Root Mean Square $\left({RMS}_{AP}\right)$

(3) $$RMS_{AP}={\left[\frac{1}{N}\sum \;AP[n{]}^{2}\right]}^{1/2}$$

ML Room Mean Square $\left({RMS}_{ML}\right)$

(4) $$RMS_{ML}={\left[\frac{1}{N}\sum \;ML[n{]}^{2}\right]}^{1/2}$$

AP Peak-to-Peak Displacement $\left({MAXD}_{AP}\right)$

(5) $${MAXD}_{AP}=max(AP)-min(AP)$$

ML Peak-to-Peak Displacement $\left({MAXD}_{ML}\right)$

(6) $$MAXD_{ML}=max(ML)-min(ML)$$

Total Excursion (TE)

(7) $$TE=\sum_{n=1}^{N-1}\;{\left[(AP[n+1]-AP[n]{)}^{2}+\left(ML[n+1]-ML[n{]}^{2}\right)\right]}^{1/2}$$

95% Area Ellipse (AE)

(8) $$AE=2\pi F_{.05[2,n-2]}{\left[s_{AP}^{2}s_{ML}^{2}-s_{APML}^{2}\right]}^{1/2}$$

where,

(9) $$s_{AP}^{2}=\left[\frac{1}{N}\sum \;AP[n{]}^{2}\right]$$

(10) $$s_{ML}^{2}=\left[\frac{1}{N}\sum \;ML[n{]}^{2}\right]$$

(11) $$s_{APML}=\frac{1}{N}\sum AP\left[n\right]ML[n]$$

#### 2.2.2. Statistical Analysis

For each parameter, both pre- and post-assessment averages and standard deviations across participants were computed (Table 2). Statistical analysis was performed using the SAS/STAT software Version 9.4 (SAS Institute Inc., Cary, NC, USA). For our normally distributed data, *t*-tests were conducted to determine statistical significance between the two groups. A *p*-value of 0.05 indicated significance. Additionally, due to the small sample size, the effect size was calculated using Cohen’s d. This measurement was calculated separately for the control and VR groups via the mean differences between the pre- and post-training means divided by the pooled standard deviation. A lower Cohen’s d indicates the necessity of larger sample sizes.

## 3. Results

Table 2 Shows the average values and standard deviations for RMS and MAXD (both AP and ML), TE and AE pre- and post-training for control and VR groups. Further, the results for the effect size calculations for both groups are shown in Table 3.

The results in Table 3 show effect size calculations using Cohen’s d equation. Cohen classified effect sizes as small (d = 0.2), medium (d = 0.5), and large (d ≥ 0.8) [27]; the larger the effect size, the more powerful it is. In this case, the effect size is a quantification of the difference between two calculated means. A smaller effect size indicates that the means between the pre- and post-training values (e.g., 0.2) only differ by 0.2 standard deviations, so the differences are trivial. A medium effect size (e.g., 0.5) indicates that the two means differ by 0.5 standard deviations, which can be considered non-trivial, but significance may be a question. A larger effect size (e.g., >0.8) signifies a larger difference between the means and is confirmed by the results of the *p*-value. The smaller Cohen’s d values reported in this study indicated the potential need for a larger sample size required to make statistically significant differences; however, medium and large Cohen’s *d* values were observed in both control and VR groups for certain parameters.

**Figure 4.**
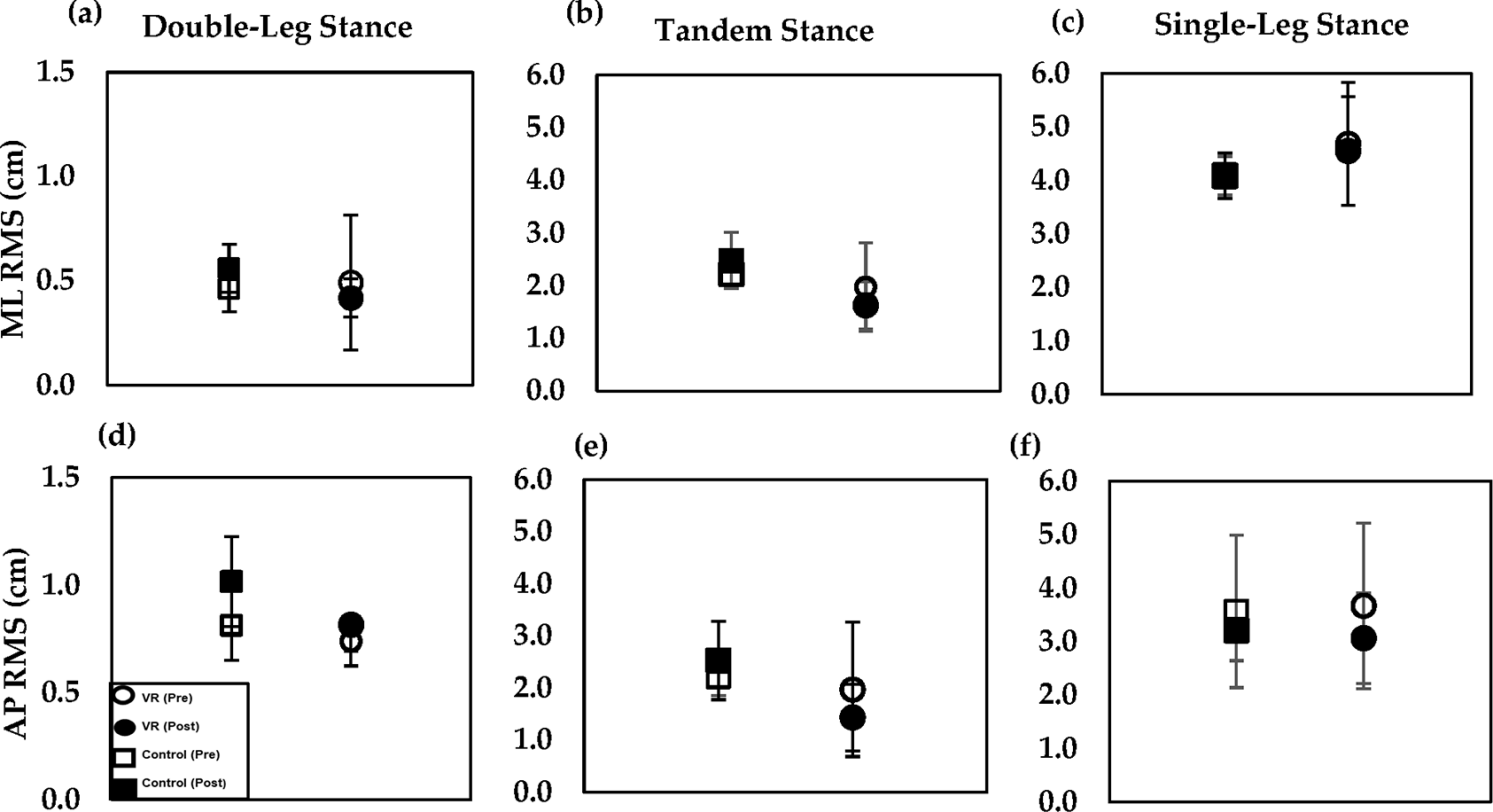
ML RMS (top): (**a**) double-leg, (**b**) tandem, and (**c**) single-leg stances; AP RMS (bottom) (**d**) double-leg, (**e**) tandem, and (**f**) single-leg stances. Results for VR group and control group pre-training (open icons) and post-training (filled icons) with standard error bars shown.

**Figure 5.**
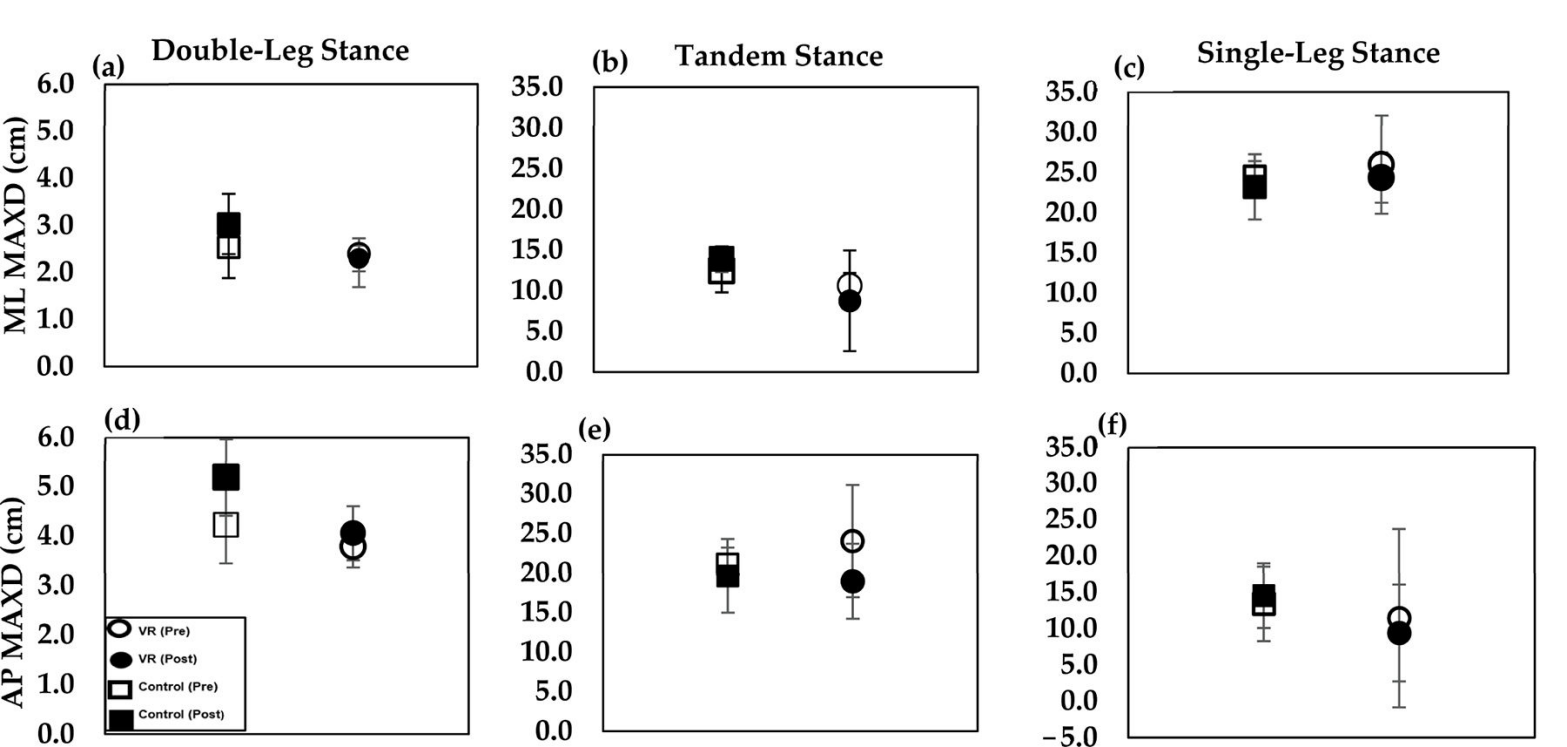
ML MAXD (top). (**a**) double-leg, (**b**) tandem, and (**c**) single-leg stance and AP MAXD (bottom) (**d**) double-leg, (**e**) tandem, and (**f**) single-leg stance. Results for VR group and control group pre-training (open icons) and post-training (filled icons) with standard error bars shown.

**Figure 6.**
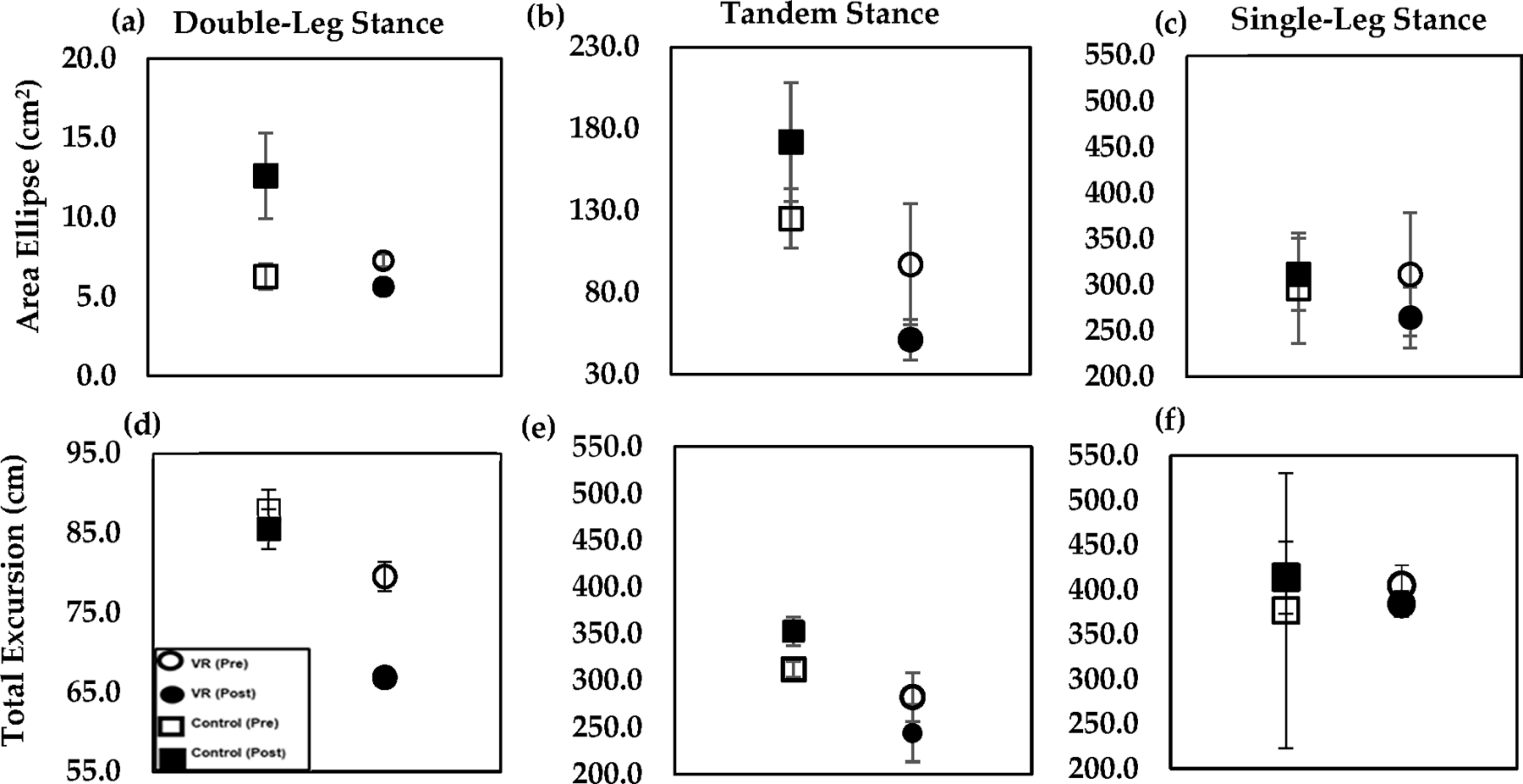
95% Area ellipse (top). (**a**) double-leg, (**b**) tandem, and (**c**) single-leg stance and total excursion (bottom) (**d**) double-leg, (**e**) tandem, and (**f**) single-leg stance. Results for VR group and control group pre-training (open icons) and post-training (filled icons) with standard error bars shown.

Graphical results are shown for the ML and AP RMS (Figure 4), ML and AP MAXD (Figure 5), TE (Figure 6a–c) and AE (Figure 6d–f), across all stance positions. Several changes in RMS ML and AP data were seen post-training compared to pre-training. Any differences seen in RMS ML and AP for the control group, pre-training compared to post-training in the double-leg, tandem, and single-leg stances, were found to be insignificant (*p* > 0.996). Furthermore, RMS ML and AP changes in the VR group in the double-leg, tandem, and single-leg stances were also found to be insignificant (*p* > 0.707).

Differences in MAXD were observed post-training compared to pre-training in both the VR and control groups. Average ML and AP MAXD changes in the control group post-training compared to pre-training in the double-leg, tandem, and single-leg stances were statistically insignificant (*p* > 0.996). Additionally, no significant differences were found comparing pre-training to post-training for AP and ML MAXD values for the VR and control groups (*p* > 0.579).

Comparison of the area ellipse pre-training to post-training in both the VR and control groups produced varied results. The VR group AE values in the double-leg, tandem, and single-legstances, were not found to be statistically significant (*p* > 0.281). The control group also did not show a significant improvement in AE resultsacross all stances (*p* > 0.996).

Comparing total excursion post-training to pre-training for both the VR and control groups also produced mixed results. The VR group TE values showed a 16% significant decrease (*p* = 0.011), but not in the tandem and single-leg stances (*p* > 0.996). However, the control group did not show a significant improvement in all three stances (*p* > 0.992).

## 4. Discussion

In this study, we aimed to investigate the impact of a VR-based training program on healthy older adults’ balance ability. VR participants reduced the combined AP and ML COP parameter, total excursion (TE), pre- compared to post-training for the double-leg stance. A reduction of this COP parameter was interpreted as the VR group decreasing the total length of the COP, and thus, they were able to maintain better balance for this test condition. Improved balance was consistent with previous studies.

Other studies reported improvements in balance after exercise training with the use of a VR device [11,14–16,21,28,29]. In a study by Duque et al., results after post-training with a VR system showed improvements in balance parameters, which included AE and maximum sway [17]. Meanwhile, another study only analysed tandem stance amongst older adults before and after VR-based exercise training [16]. Both studies reported an improvement in standing balance post-VR exercise training compared to pre-training. A review study also researched the effectiveness of immersive VR training on balance, gait, and mobility, reporting that VR training can improve balance in older adults [15,20,21]. However, studies have reported inconsistent results (either improvement or no improvement) between control and VR participants post-exercise training. One study reported no significant changes in balance ability pre- to post-exercise training for both the control and VR groups [30]. Another study reported similar changes in balance post-exercise training for both the VR and control groups, signifying no method was superior to the other. Additionally, other studies have commonly only reported on the TUG or the Falls Efficacy Scale as assessment measures; however, these do not identify the details of an individual’s postural movements that may lead to an increase in fall risk [15,20,21]. Therefore, our study provides more concrete metrics (COP parameters) to capture and analyze an individual’s balance improvement.

For the control participants, we also observed insignificant changes in the RMS AP, MAXD AP and ML, and in TE. The control group had a worsening (increase) of the area ellipse (AE) for the double-leg stance. While this study did not see significant improvement in balance ability from the control group, a related study reported that the control group had enhanced balance and postural control after a 6-week exercise training course [15]. Further, one important factor to note is, on average, the control group had poorer performance compared to the VR group pre-training, which may have led to mixed differences post-training.

Overall, our study’s analysis of the COP parameters post-training could advise the progression of targeted interventions specific to areas needing balance improvement in older adults. Additionally, recognizing the success of exercise training on postural stability and balance ability allows clinical personnel to tailor rehabilitation strategies, specifically for older adults who may be at risk for falls. Our results showed variability in postural stability outcomes. Lastly, this study contributes to the developing list of research on VR use, exercise training, and balance performance. To continue to improve future research, comprehending these aspects is critical to adapting intervention plans and improving outcomes aimed to improve balance and decrease the risk of falls in older adults. The use of VR training may provide an added benefit outside of the traditional exercise regimen, such as improved incentive and enjoyment leading to increased compliance with prescribed exercise training among older adults. However, more research is needed on adherence and useability [31].

### Limitations

In our study, there were some potential limitations. The differences in outcomes found from significance testing for the COP parameters across all stances signifies the multifaceted approach to assessing postural stability. These differences were seen in total excursion and 95% area ellipse measures. The sample size of this study may be a limiting factor in terms of the significance of findings. However, the smaller sample size employed in this study could still offer notable results when procedures are carefully selected, and data sets thoroughly analyzed. Yet, a larger sample size could improve the vigor and reliability of the results and substantiate significant differences. We were bound by the number of participants due to time constraints and recruitment of participants within a set timeframe. Moreover, the individual differences between each of the participants, including exposure to other exercise programs and knowledge of the exercises in this study, may have contributed to the disproportions reported in the results. Each participant’s approach during the collection of data post-assessment could have been influenced by several factors, namely, their prior experience with the assessment protocol and comfort with specific exercises. Additionally, other factors may have included exhaustion, motivation, and attention to detail.

## 5. Conclusions

Overall, our study’s analysis of COP parameters post-training could advise the progression of targeted interventions specific to areas needing balance improvement in older adults. Additionally, recognizing the success of exercise training on postural stability and balance ability allows clinical personnel to tailor rehabilitation strategies, specifically for older adults who may be at risk for falls. Our results also showed variability in postural stability outcomes. Lastly, this study contributes to the developing list of research on VR use, exercise training, and balance performance. To continue to improve future research, comprehending these aspects is critical to adapting intervention plans and improving outcomes aimed to improve balance and decrease the risk of falls in older adults.

## Figures and Tables

**Table 1.** Overview of participant demographics (gray text indicates withdrawal from the study) [13].

			Medical Conditions					Physical Abilities				Visual Abilities	
Subject	Age	Gender	Ethnicity	Traumatic Brain Injury	Dizziness/Vertigo	Other	Previous Falls	Meds Cause Dizziness/Loss of Balance	Exercise	Physical Limitations	Perceived Fitness Level (10 = most, 1 = least)	Glasses	Vision Loss
VR1	73	M	Caucasian	No	No	LTKR, lumbar spinal fusion	No	Yes	Yes (bike, swim, weights, stretch)	No	10	Yes	No
VR2	82	F	Caucasian	No	No	No	No	Y (Lunesta)	Yes (4×/wk, walk, yoga, weights)	No	10	No	No
VR3	78	F	Caucasian	No	No	Arthritis (hips/back)	No	No	NA	NA	NA	NA	NA
VR4	76	F	Caucasian	No	No	No	No	No	Yes (walk, weights, stretch)	No	6	Yes	No
VH5	77	F	Caucasian	No	No	No	No	No	Yes(walking)	Left shoulder issues	7	Yes	No
VR6	72	F	Black/African American	No	No	No	Yes (trip and fall of step)	No	Yes (Bike 6×/wk, strength and stretch 2×)	No	6	Yes	No
VR7	NA	F	Caucasian	NA	NA	NA	NA	NA	NA	NA	NA	NA	NA
VR8	78	F	Caucasian	No	No	Mid-low back if sit too long	No	No	Yes (walk, 10 k steps/day, sometimes bike)	No	6	Yes	No
VR9	71	M	Caucasian	No	No	No	No	Yes (metformin, metoprolol)	Yes (6–7 days/wk of walking)	No	7	No	No
C1	78	F	Caucasian	No	No	No	No	No	Yes (walk several×/wk)	No	6	Yes	No
C2	79	F	Caucasian	No	No	Osteoporosis	Yes (once 2 month ago)	No	Yes (2×/wk)	NA	6	Yes (post-cataract surgery)	No
C3	82	F	Caucasian	No	No	No	Yes (on ice 3 years ago)	No	Yes (bike, walk, weights)	No	9	Yes (post-cataract surgery)	No
C4	80	F	Caucasian	No	No	No	Yes (trip and fall while walking)	No	Yes (walk, weights, bike)	No	10	No (cataract surgery 5 years ago	No
C5	78	F	Caucasian	No	No	No	No	No	(Yes, walking, bike)	No	6	Yes	No
C6	62	F	Black/African American	No	Yes	Osteoarthritis	No	No	Yes (5 + days, pool/other)	No	7	Yes	No
C7	73	F	Black/African American	No	No	No	No	No	Yes(5×/wk)	NA	6	NA	No
C8	69	F	Caucasian	No	No	No	No	No	Yes (4–5 days/wk, boxing, swimming, gym workout)	No	9	Yes	No
W	78	F	Moroccan	NA	No	No	No	No	Yes (walking)	NA	NA	Yes	No
W	72	F	Caucasian	No	No	No	Yes (trip and fall off step)	No	Yes (Bike 6×/wk, strength and stretch 2×/wk)	No	6	Yes	No
W	NA	F	Black/African American	NA	NA	NA	NA	NA	NA	NA	NA	NA	NA
W	83	M	Caucasian	No	No	NA	Yes (trip, broke nose, 1 month ago)	No	No (last time 2 years ago)	No	4	Yes	No

**Table 2.** Root mean square, peak-to-peak displacement, total excursion, and area ellipse for control (*n* = 8) and VR (*n* = 9) participants pre- and post-exercise training. Means and standard deviations (in parentheses) are shown.

		Control						VR					
		Pre			Post			Pre			Post		
		Double	Tandem	Single	Double	Tandem	Single	Double	Tandem	Single	Double	Tandem	Single
RMS (cm)	ML	0.46(0.11)	2.22(0.27)	*4.09(0.37)*	0.56(0.11)	2.48(0.52)	4.09(0.42)	0.49(0.32)	1.97(0.84)	4.68(1.1)	0.42(0.09)	1.62(0.44)	4.55(1.0)
	AP	0.81(0.17)	2.22(0.37)	*3.56(1.4)*	1.02(0.21)	2.53(0.69)	3.21(0.57)	0.74(0.11)	1.97(1.3)	3.67(1.5)	0.82(0.11)	1.43(0.63)	3.06(0.85)
MAXD (cm)	ML	2.53(0.65)	12.4(1.9)	*24.5(2.6)*	3.03(0.64)	13.9(4.0)	23.2(1.6)	2.40(0.33)	10.6(6.1)	26.0(6.0)	2.29(0.67)	8.74(3.0)	24.3(6.0)
	AP	4.22(0.78)	13.4(2.1)	*21.1(5.1)*	5.19(0.78)	14.5(4.7)	19.6(4.5)	3.79(0.42)	11.4(7.1)	24.0(11)	5.31(6.2)	9.38(4.6)	18.9(6.4)
AE (cm^2^)		6.30(2.3)	125(51)	*296(170)*	12.6(6.4)	172(102)	265(84)	5.51(1.2)	97.2(107)	311(165)	5.62(1.4)	51.4(35)	238(93)
TE (cm)		87.8(7.3)	312(24)	*377(18)*	85.5(5.8)	353(43)	414(51)	79.5(5.8)	283(75)	405(65)	66.8(3.4)	244(40)	384(40)

Significance: 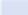 *p* < 0.05, *n* = 6 (two participants (in italics) opted not to do single-leg stance pre-training due to fear of instability).

**Table 3.** Cohen’s *d* results for both the control and VR groups. Small (red), medium (blue), and large (green) effect sizes are noted in the table; negative symbol shown to indicate directionality.

		Control			VR		
		Cohen’s d			Cohen’s d		
		Double	Tandem	Single	Double	Tandem	Single
RMS (cm)	ML	0.51	0.16	−0.19	0.32	−0.46	−0.64
	AP	0.53	0.16	−0.04	−0.41	−0.42	−0.07
MAXD (cm)	ML	0.29	0.11	−0.09	0.26	−0.31	−0.62
	AP	0.44	0.14	−0.13	−0.13	−0.37	−0.40
AE (cm^2^)		0.79	0.19	−0.14	0.04	−0.63	−0.73
TE (cm)		−0.08	0.20	−0.06	−0.77	−0.29	0.20

## Data Availability

The data presented in this study are available on request from the corresponding author.
